# Potential risk of tamoxifen: gut microbiota and inflammation in mice with breast cancer

**DOI:** 10.3389/fonc.2023.1121471

**Published:** 2023-07-04

**Authors:** Hailong Li, Xiufei Gao, Yian Chen, Mengqian Wang, Chuchu Xu, Qinghong Yu, Ying Jin, Jiaqing Song, Qi Zhu

**Affiliations:** ^1^ School of Green Intelligent Pharmaceutical Industry, Zhejiang Guangsha Vocational and Technical University of Construction, Dongyang, Zhejiang, China; ^2^ Department of Breast Surgery, The First Affiliated Hospital of Zhejiang Chinese Medical University (Zhejiang Provincial Hospital of Chinese Medicine), Hangzhou, Zhejiang, China; ^3^ First Clinical Medical College, Zhejiang Chinese Medical University, Hangzhou, Zhejiang, China

**Keywords:** breast cancer, gut microbiota, TLR5, inflammation, tamoxifen

## Abstract

**Objective:**

Tamoxifen is an effective anti-tumor medicine, but evidence has been provided on tamoxifen-related inflammation as well as its impact on gut microbiota. In this study, we aimed to investigate tamoxifen-induced gut microbiota and inflammation alteration.

**Methods:**

We established a BC xenograft mouse model using the MCF-7 cell line. 16S rRNA gene sequencing was used to investigate gut microbiota. qRT–PCR, western blotting, and cytometric bead array were used to investigate inflammation-related biomarkers. Various bioinformatic approaches were used to analyze the data.

**Results:**

Significant differences in gut microbial composition, characteristic taxa, and microbiome phenotype prediction were observed between control, model, and tamoxifen-treated mice. Furthermore, protein expression of IL-6 and TLR5 was up-regulated in tamoxifen-treated mice, while the mRNA of Tlr5 and Il-6, as well as protein expression of IL-6 and TLR5 in the model group, were down-regulated in the colon. The concentration of IFN-γ, IL-6, and IL12P70 in serum was up-regulated in tamoxifen-treated mice. Moreover, correlation-based clustering analysis demonstrated that inflammation-negatively correlated taxa, including *Lachnospiraceae-UCG-006* and *Anaerotruncus*, were enriched in the model group, while inflammation-positively correlated taxa, including *Prevotellaceae_UCG_001* and *Akkermansia*, were enriched in the tamoxifen-treated group. Finally, colon histologic damage was observed in tamoxifen-treated mice.

**Conclusion:**

Tamoxifen treatment significantly altered gut microbiota and increased inflammation in the breast cancer xenograft mice model. This may be related to tamoxifen-induced intestinal epithelial barrier damage and TLR5 up-regulation.

## 1 Introduction

Breast cancer (BC) is the most commonly diagnosed malignant tumor worldwide, with the highest incidence rate. The burden of BC is expected to continue to increase (1). Tamoxifen is a selective estrogen receptor modulator that competitively inhibits the binding of estradiol to estrogen receptors, thereby preventing the receptor from binding to the estrogen-response element on DNA, resulting in a reduction in DNA synthesis and cellular response to estrogen. Tamoxifen has been used for many years in treating hormone receptor-positive BC (2), and receptor-negative BC patient cannot profit from tamoxifen treatment (3). However, some studies have reported that tamoxifen is associated with increased inflammation and alterations in gut microbiota (4, 5). Furthermore, conflicting evidence has been provided on whether tamoxifen therapy increases the risks of receptor-negative contralateral breast cancer (6, 7). It is not clear whether tamoxifen-related inflammation and gut microbiota alterations are associated with BC.

It has been reported that bacteria in the gastrointestinal tract are ten times the number of cells in a human body (8), and the host-microbes homeostasis is associated with inflammation repression, metabolism, intestinal permeability, etc. (9–12). Dysbiosis may lead to various diseases, including cancer. Studies have identified significant differences in gut microbiota in BC patients compared with those in healthy people (13). Emerging evidence indicates that gut microbiota affects the response to anticancer therapies by modulating the host immune system (14). Several *in vivo* and *in vitro* studies have provided remarkable evidence that diet, probiotics, and prebiotics could exert important anticarcinogenic effects in BC (15). Moreover, the community structure and function of gut microbiota can be altered in response to changes in diet, physiology, or drug intake (16, 17). Therefore, gut microbiota may be a noninvasive target for BC treatment.

This study aims to interrogate the gut microbiota and inflammation changes of tamoxifen-treated BC to identify tamoxifen-related gut microbiota and inflammation alterations. Our findings offer new evidence that tamoxifen is an inflammation promoter and GM regulator, broadening our understanding of the inflammation-GM correlation and providing new targets to reduce tamoxifen-related side effects.

## 2 Materials and methods

### 2.1 Establishment of the xenograft mice model of breast cancer

Female nude mice of Specific Pathogen-Free (SPF) level (BALB/c-nude; 18-22g, 6-8 weeks old) were purchased from Shanghai SLAC Laboratory Animal Company (Shanghai, China). The mice were housed in the animal lab of Zhejiang Traditional Chinese Medicine University and maintained under SPF level conditions at a temperature of 22-26°C and 12-hour light/dark cycle. Animal experiments were approved by the Institutional Animal Care and Use Committee of Zhejiang Traditional Chinese Medicine University. Human MCF-7 cells were purchased from the Cell Bank of Chinese Academy of Sciences of Shanghai (Shanghai, China).

All mice were kept in the animal facilities for one week of acclimation, after which the mice were randomly and equally divided into 2 groups (n=5 and 15 in each group). In the n=15 group, estrogen sustained-release tablets were planted, and 1 × 10^7^ MCF-7 cells in 0.2 mL phosphate buffer saline were injected into the left 5th mammary fat pad of the mice. After the xenograft models were build, the n=15 group was randomly divided into model group (model, n=10) and tamoxifen group (TAM, n = 5). The mice in group TAM were garaged everyday by tamoxifen solution of 100 μL/10g weight in the concentration of 4 mg/kg, and the mice in Model were administered vehicle. The dose of tamoxifen in this study was estimated based on body surface area. The clinically recommended dose of tamoxifen is 20 mg qd, for a 60 kg human, the dose is 0.33 mg/kg. It is reported that to convert human dose(mg/kg) to mouse dose, multiply human dose by 12.3

All mice were kept in the animal facilities for one week to acclimate. They were then randomly divided into two groups (n=5 and 15 in each group). In the n=15 group, estrogen-sustained release tablets were implanted, and 1 × 10^7^ MCF-7 cells in 0.2 mL phosphate buffer saline were injected into the left fifth mammary fat pad of the mice. After the xenograft models were established, the n=15 group was randomly divided into a model group (n=10) and a tamoxifen group (n=5). The mice in the tamoxifen group were orally administered tamoxifen solution of 100 μL/10g weight at a concentration of 4 mg/kg, once a day. The mice in the model group were administered vehicle. The dose of tamoxifen in this study was estimated based on body surface area. The clinically recommended dose of tamoxifen is 20 mg qd for a 60 kg human, which corresponds to a dose of 0.33 mg/kg. It is reported that to convert human dose(mg/kg) to mouse dose, the human dose should be multiplied by 12.3 (18); thus, the dose for mice is approximately 4mg/kg in this study. The mice that did not receive MCF-7 cells injection were set as the control group (n = 5) and were also given the same amount of water. All mice were allowed free access to standard food and water during the procedure, which lasted for 28 days. At the end of the procedure, fecal samples were collected from each mouse, immediately quenched in liquid nitrogen, and then stored at -80°C until DNA extraction. After that, the mice were sacrificed, and their tumor, colon, and serum were stored at -80°C for further measurement.

### 2.2 16S rRNA gene sequencing

DNA was extracted using the Fast DNA^®^ Spin Kit for Feces (MP Biomedicals, California, USA) following the recommended protocol. The V3–V4 region of the 16S rRNA gene was amplified with a S100 thermal cycler (Bio-Rad Laboratories, USA) following the standard protocol: 95°C for 2 minutes, 30 cycles at 95°C for 30 seconds for denaturation, 52°C for 30 seconds for annealing, 72°C for 30 seconds for extension, and a final extension at 72°C for 7 minutes. Forward primers 341F (5′-ACTCCTACGGGRSGCAGCAG-3′) and reverse primers 806R (5′-GGACTACVVGGGTATCTAAT-3′) were used. Sequencing of the 16S rRNA gene was performed using an Illumina NovaSeq PE250 (Illumina, CA, USA). The raw data were quality-controlled using DADA2, and the resulting high-quality reads were clustered to generate features at 100% similarity. Identification and annotation were carried out using the SILVA 16S rRNA database (http://www.arb-silva.de) and NT-16S, and alpha diversity (Shannon, Simpson, and Chao1) was calculated using Qiime 2. Rarefaction curves were analyzed using Mothur, and the LEfSe algorithm was employed with the nonparametric factorial Kruskal Wallis test (α = 0.05).

### 2.3 Cytometric bead array

Cytokine assessment was carried out using mice inflammation CBA kit (BD Bioscience, San Jose, USA) for simultaneous detection of six cytokines (IL-6, IL-10, MCP-1, IFN-γ, TNF-α and IL-12p70) in plasma diluted (1/10) with appropriate diluent.

Cytokines were determined in the test samples according to the manufacturer instructions. Briefly, test samples (50 ll) and PE detection antibody were incubated with capture bead reagent for 3 h in dark at room temperature. All unbound antibodies were washed (1.0 ml wash buffer) and re-suspended in 300 ll before acquisition on BD Accuri™ C6 Plus Flow Cytometer (BD Bioscience, San Jose, CA, USA). All six cytokines exhibited single and well separated peaks. Six individual cytokine standard curves (range 20–5000 pg/ml) were run in each assay. The range of detection was between 3 and 5000 pg/ml calculated from curve estimation for an average of five assays using power fit and R2 > 0.99 for all cytokines. Inter and intra assay coefficients of variation for all cytokines were described by the manufacturer in the instruction manual. To establish the contribution of plasma cytokines, culture supernatants and plasma were compared at equivalent dilutions.

### 2.4 Western blotting

Before blotting, the protein was quantified using the bicinchoninic acid method. Simple Western immunoblotting was performed on a Simple Wes System (ProteinSimple, CA, US) using a Size Separation Master Kit with Split Buffer (12–230 kDa) based on the manufacturer’s standard instruction and using anti–β-actin antibodies (cell signaling technology, Danvers, US). Compass software (version 4.0.0, ProteinSimple) was used to program the Simple Wes and present (and quantification) the Western immunoblots. Output data were displayed from the software calculated average of seven exposures (5–480 s).

### 2.5 qRT-PCR

Total RNA was extracted using TRIzol reagent (Invitrogen) following the manufacturer’s instructions, and quantitative real-time polymerase chain reaction (qRT-PCR) was performed according to previous work (Wu, Qiu et al., 2018). The qRT-PCR was performed on a LightCycler 480 (Roche Diagnostics) using SYBR Green Master Mix (Roche). The primer information is shown in **Supplementary Table S1**. The relative quantitative analysis of mRNA levels was calculated using the 2^-ΔΔCt^ method.

### 2.6 Intestinal mucosal membrane histology

Histological sections of the colon tissue were obtained as previously described (19). The mucosal membrane was considered damaged if there was evidence of shortened villi or increased inflammatory cell infiltration. Eight random fields were assessed for each mouse.

### 2.7 Statistical methods

The results were presented as means ± SEM. Statistical analyses were conducted using SPSS 19.0 statistical software (SPSS, USA). One-way ANOVA and Kruskal-Wallis tests were utilized for multi-group comparisons. Mann-Whitney U test was employed for comparisons between two groups, while Kolmogorov-Smirnov test was used for comparisons of mRNA between two groups. Correlations between two parameters were evaluated using the Spearman correlation test. Data visualization was performed using GraphPad Prism 8.0. A P-value of <0.05 was considered statistically significant.

## 3 Results

### 3.1 Tamoxifen significantly inhibited tumor growth in MCF-7 xenografts

As previously reported, tamoxifen demonstrated a significant inhibitory effect on MCF-7 xenograft tumor growth in nude mice after 28 days of treatment (20).

### 3.2 Significant differences in gut microbiota among the three groups

**Figure 1:**
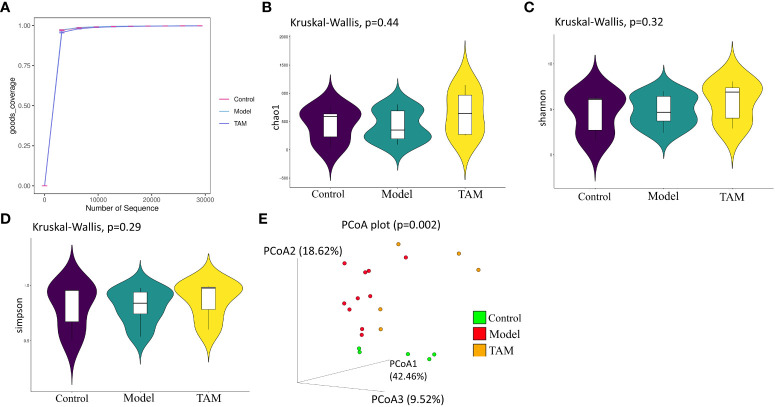
Gut microbiome community diversity analysis. Species α-diversity index and statistical significance among three groups were estimated by **(A)** goods coverage index; **(B)** Chao1 index; **(C)** Shannon index; **(D)** Simpson index. Additionally, **(E)** Species β-diversity differences among three groups were estimated by PCoA plot analysis of weighted UniFrac. Control group (green dots); model group (red dots); TAM group (yellow dots), where dots represent individual samples. n = 5 for control group, n = 10 for model group and n = 5 for TAM group.

In this study, 16S rRNA gene sequencing was used to analyze 20 fecal samples from the three groups, identifying 20 phyla and 295 genera. **Figure 1A** shows the sequencing depth evaluated by rarefaction curves based on the goods coverage index, which indicates the adequacy of the sampling effort. Significant differences were observed in the gut microbiota among the three groups.

#### 3.2.1 Community diversity

Microbial richness was estimated using the α-diversity index Chao 1 (**Figure 1B**), and evenness was estimated using the Shannon (**Figure 1C**) and Simpson indices (**Figure 1D**). There were no significant differences in microbial α-diversity among the three groups (p>0.05), although the model group exhibited lower Chao1, Shannon, and Simpson indices compared to the control and TAM groups. It has been reported that the α-diversity Shannon index is lower in breast cancer patients before any treatment (21). In the principal coordinates analysis (PCoA) of weighted UniFrac distances (**Figure 1E**), the three groups showed a trend of separation based on the first three PCoA. The variance of the first two principal component scores was 42.46% and 18.62%, respectively (p=0.002). These results suggest significant alterations in gut microbiota in the model group as well as the TAM group, which may be induced by estrogen and estrogen+ tamoxifen treatment, respectively.

#### 3.2.2 Microbial composition

**Figure 2:**
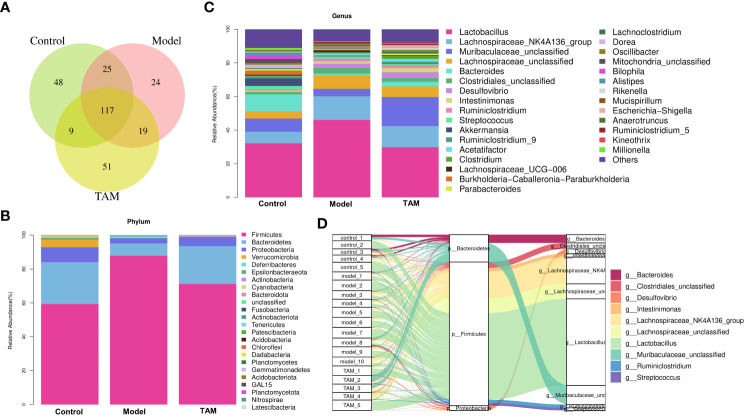
Microbial composition analysis. **(A)** Venn diagram shows shared genera in control, model and TAM groups. **(B)** Community abundance bar plot on phylum level of 3 groups. **(C)** Community abundance bar plot on genus level of 3 groups. **(D)** Sankey diagram shows relative abundance of each sample on phylum and genus level.

As shown in **Figure 2A**, 199, 185, and 196 genera were identified in the control, model, and TAM groups, respectively, with 117 genera shared by all 3 groups, 142 shared between the model and control groups, 136 shared between the model and TAM groups, and 126 shared between the control and TAM groups. Furthermore, considerable variability in microbial composition was observed across samples in each group.

The relative proportions of taxa at the phylum and genus levels were assessed. At the phylum level, the gut microbiota in all groups were dominated by three phyla: Bacteroidetes, Firmicutes, and Proteobacteria. The relative abundance of Firmicutes in the model group was higher than that in the control and TAM groups, while Bacteroidetes were lower. The cumulated relative abundances of these three phyla were 93.00-99.39% in the three groups (**Figures 2B, D**).

At the genus level, *Lactobacillus, Lachnospiraceae_NK4A136_group, Muribaculaceae_unclassified*, *Lachnospiraceae_unclassified, Bacteroides, Clostridiales_unclassified, Desulfovibrio, Intestinimonas, Ruminiclostridium*, and *Streptococcus* were identified as the top 10 dominated genera (**Figures 2C, D**).

**Figure 3:**
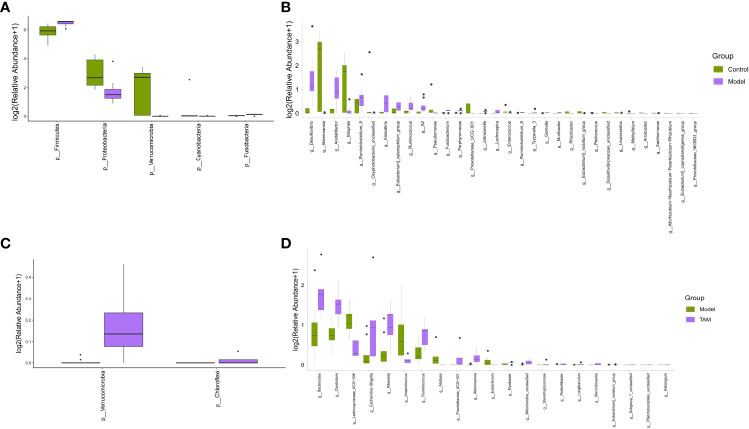
Significant difference in microbial composition. Differential abundance bar plot on **(A)** phylum level between control and model groups; **(B)** genus level between control and model groups; **(C)** phylum level between model and TAM groups; and **(D)** genus level between model and TAM groups.

A Mann-Whitney U test was conducted to identify genera and phyla with significant differences in relative abundance between the control group and model group, as well as between the model and TAM groups. As shown in **Figures 3A, B**, compared to the control group, the phylum Firmicutes and genera *Desulfovibrio, Acetatifactor, Ruminiclostridium_5, Kineothrix, Eubacterium_xylanophilum_group, Ruminococus*, and *A2* were significantly up-regulated, whereas the phyla Proteobacteria and Verrucomicrobia and genera *Akkermansia* and *Bilophila* were significantly down-regulated in the model group (**Figures 3A, B**). Interestingly, a higher abundance of Firmicutes was previously reported in BC patients (21). Compared to the model group, the phyla Verrucomicrobia and Chloroflexi and genera *Bacteroides, Clostridium, Escherichia-Shigella, Ruminococcus, Prevotellaceae_UCG-001*, and *Akkermansia* were significantly up-regulated, whereas genera *lachnospiraceae_UCG-006, Anaerotruncus, Alistipes*, and *Eubacterium* were significantly down-regulated in the TAM group (**Figures 3C, D**).

**Figure 4:**
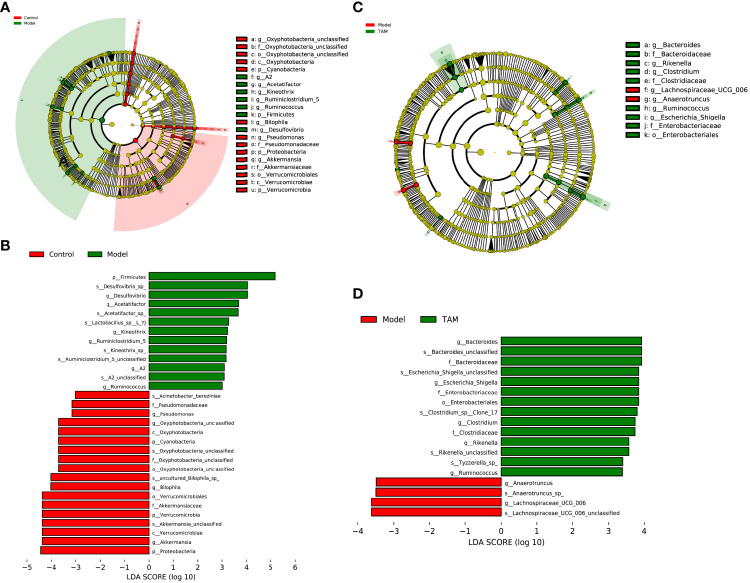
Linear discriminant analysis (LDA) integrated with effect size (LEfSe). **(A)** Cladogram indicating the phylogenetic distribution of microbiota correlated with control or model group. **(B)** The differences in abundance of LDA score between control and model group. **(C)** Cladogram indicating the phylogenetic distribution of microbiota correlated with model or TAM group. **(D)** The differences in abundance of LDA score between model and TAM group.

Considering that the discriminant analysis did not distinguish the predominant taxon, LEfSe was used to identify the characteristic taxa of each group. A LDA score > 3 was used to identify the statistically significant representative types. As shown in **Figures 4A, B**, 18 and 13 representative taxa were identified for the control and model groups, respectively. For the model and TAM groups, 4 and 14 representative taxa were identified, respectively (**Figures 4C, D**).

In the control group, *Akkermansia, Bilophila, Oxyphotobacteria_unclassified, Pseudomonas*, and *Prevotellaceae_UCG_001* were identified as characteristic genera. In the model group, *Desulfovibrio, Acetatifactor, Ruminiclostridium_5, Kineothrix, Eubacterium_xylanophilum_group, Ruminococcus, A2, Lachnospiraceae_UCG_006, Anaerotruncus, Alistipes*, and *Eubacterium* were identified as characteristic genera. In the TAM group, *Bacteroides, Clostridium, Escherichia_Shigella, Rikenella, Ruminococcus, Prevotellaceae_UCG_001, Muribacter*, and *Mitochondria_unclassified* were identified as characteristic genera.

#### 3.2.3 Microbiome-interaction patterns

**Figure 5:**
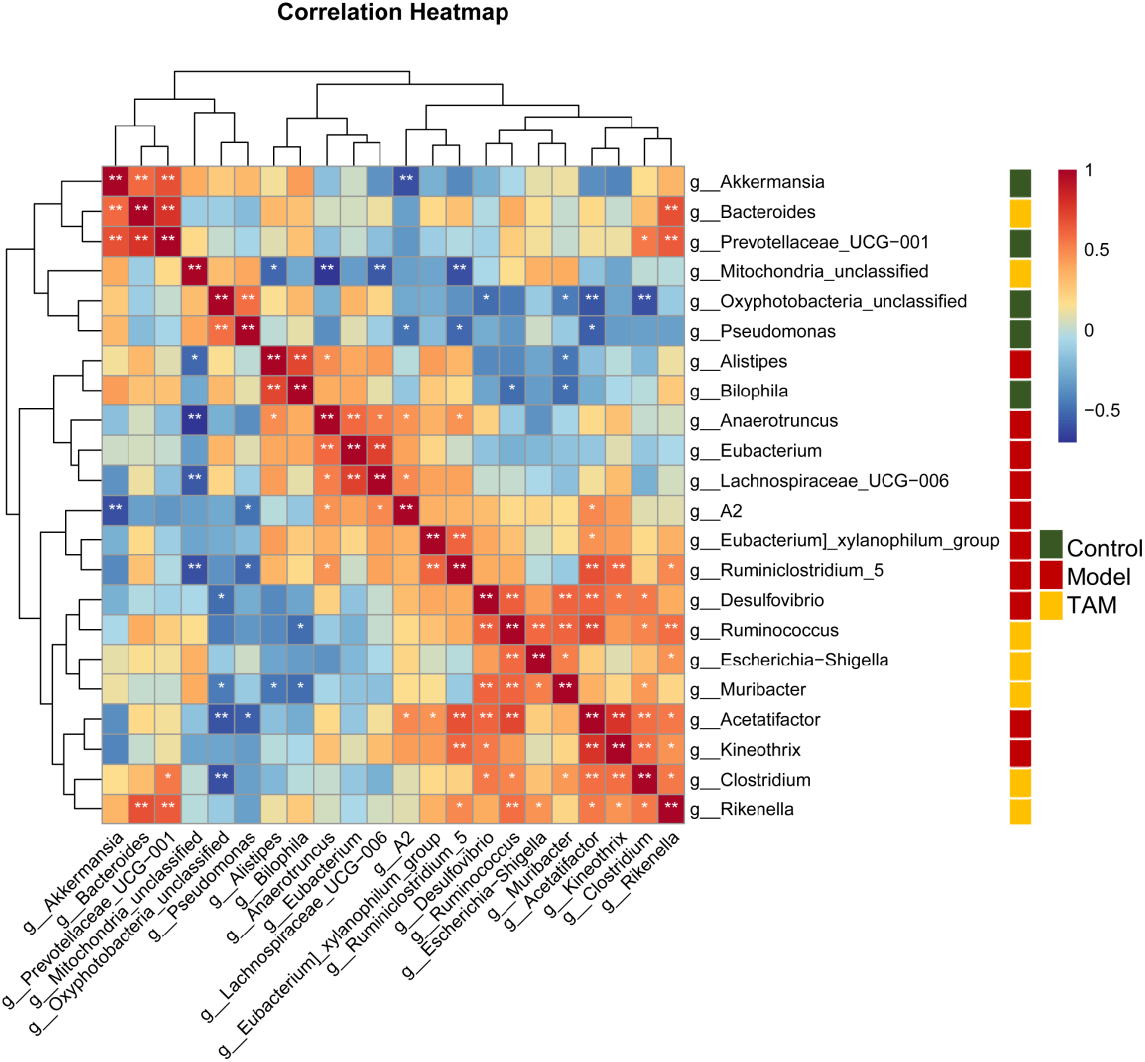
Heat map based on correlation clustering analysis of the identified characteristic genera. Genera are shown from negative correlation (in blue) to positive correlation (in red). Control group characterized (green); model group characterized (red); TAM group characterized (yellow). ∗*P* < 0.05; ∗∗*P* < 0.01.

Based on the sequencing data mentioned above, a Spearman’s correlation-based clustering analysis of the identified characteristic genera was performed to identify microbiome-interaction patterns (refer to **Figure 5**). It was observed that genera representing different groups tended to cluster into separated regions from each other, and genera representing the same group tended to cluster together.

#### 3.2.4 Microbiome function prediction

**Figure 6:**
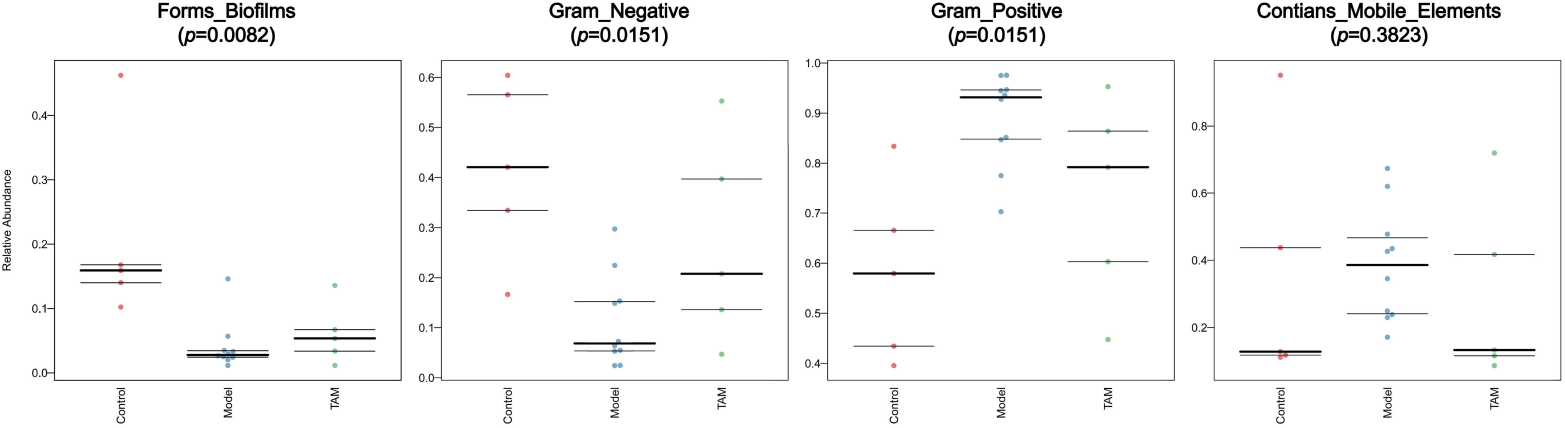
Predicted microbiome phenotype abundance and statistical significance of Form_Biofilms, Gram_Negative, Gram_Positive phenotype among 3 groups.

Investigating the functional phenotypes from microbial samples is essential in understanding the impact of gut microbiota alterations on interaction and homeostasis with the host (22). To further explore the functional differences of gut microbiota between the three groups, microbiome phenotype prediction was carried out using Bugbase (https://bugbase.cs.umn.edu). Significant differences were found in the Form_Biofilms, Gram_Negative, and Gram_Positive phenotypes between the groups. The results indicated that the gut microbiota in the model and TAM groups, especially in the model group, had a decreased ability to form biofilms and a decreased gram-negative/positive ratio compared to that in the control group. Furthermore, although not significant, the phenotype “Contain Mobile Elements” showed a higher level of mobile elements in the model group than that in the other groups (refer to **Figure 6**).

### 3.3 Increased inflammation in TAM group and decreased inflammation in model group in mice’s colon and serum

**Figure 7:**
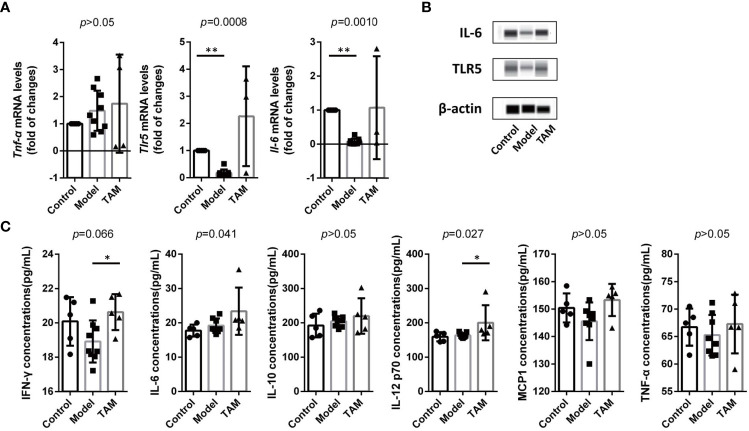
Inflammation is decreased in model group and increased in TAM group. **(A)** mRNA level and statistical significance of *Tlr5, Il-6*, and *Tnf-α* in colon tissue. **(B)** IL-6 and TLR5 expression in colon tissue. **(C)** Concentration and statistical significance of IFN-γ, MCP-1, IL-6, IL-10, TNF-α and IL12P70 in serum. Data are expressed as mean ± SEM. ∗*P* < 0.05; ∗∗∗*P* < 0.0005.

To investigate inflammation levels in mice’s colon, qRT-PCR was used to measure mRNA expression of *Tlr5, Il-6*, and *Tnf-α* in colon tissues. Compared to the other groups, mRNA expression of *Tlr5* and *Il-6* was down-regulated in the model group when compared to the control group (**Figure 7A**). Additionally, WB was performed to measure protein expression of IL-6 and TLR5 in colon tissues, and the results showed elevated IL-6 and TLR5 in all groups, but lower levels in the model group (**Figure 7B**).

To further examine systemic inflammation biomarkers, cytokines related to inflammation, including IFN-γ, MCP-1, IL-6, IL-10, TNF-α, and IL-12P70 in serum, were analyzed using CBA (**Figure 7C**). Although only IFN-γ, IL-6, and IL-12P70 showed significant differences, all the aforementioned biomarkers were elevated in the TAM group. These results indicate an increased inflammation level in the TAM group and a lower inflammation level in the model group.

### 3.4 Correlation between representative taxa and inflammation

**Figure 8:**
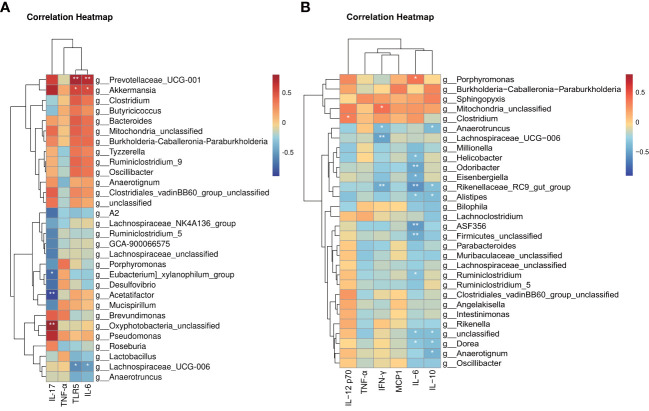
Heat map based on integrated correlation clustering analysis of gut microbiota and **(A)** mRNA level of *Tlr5, Il-6* and *Tnf-α* in colon tissue; **(B)** protein level of IFN-γ, MCP-1, IL-6, IL-10, TNF-α and IL12P70 in serum. Correlations are shown from negative correlation (in blue) to positive correlation (in red). ∗∗*P* < 0.05; ∗∗*P* < 0.01.

Our previous findings identified characteristic taxa of each group and confirmed increased inflammation in the TAM group. However, whether these characteristic taxa are correlated with inflammation remained unclear. To examine this further, we performed correlation analysis between individual microbial sample data (regardless of their group) and inflammation biomarkers. Kendall’s correlation-based clustering analysis was performed to identify microbe-inflammation associations. We included all inflammation biomarkers in colon and the top 60 abundant genera, while showing the top 30 correlated genera (**Figure 8**).

Notably, these data only suggest a potential correlation between gut microbes and inflammation biomarkers without considering the reason for these correlations. The results revealed several significantly correlated microbe-inflammation pairs. *Lachnospiraceae_UCG_006* and *Anaerotruncus*, characteristic genera of the model group, were negatively correlated with most inflammation biomarkers we included, whereas *Prevotellaceae_UCG_001, Akkermansia, Bacteroides, Clostridium*, and *Mitochondria_unclassified*, characteristic genera of the control and TAM groups, were positively correlated with most inflammation biomarkers.

Furthermore, mRNA levels of *Tnf-α* and serum levels of TNF-α showed weaker correlation with gut microbiota than other biomarkers. mRNA levels of *Tlr5, Il-6*, and serum levels of IL-12 p70 and MCP1 showed stronger positive correlation with characteristic genera of the control and TAM groups, while serum levels of IFN-γ, IL-6, and IL-10 showed stronger negative correlation with characteristic genera of the model group.

### 3.5 Effect of tamoxifen on the mucosal morphology of colon tissue in mice

**Figure 9:**
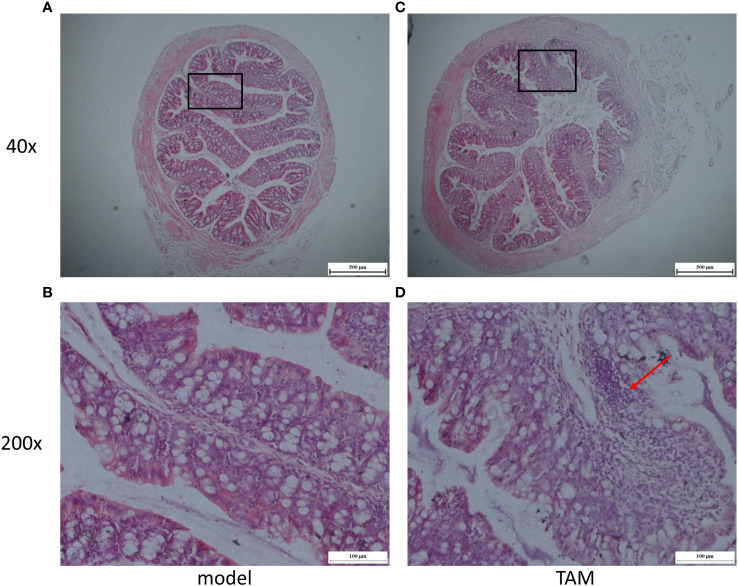
Representative micrographs of **(A)** model group colon segment, Magnification is ×40; **(B)** model group colon segment, Magnification is ×200; **(C)** TAM group colon segment, Magnification is ×40; **(D)** TAM group colon segment, Magnification is ×200. Arrow shows evidence of inflammation.

To further investigate the effect of tamoxifen on the intestinal barrier function in mice with breast cancer, we analyzed the pathological sections of colon tissues through H&E staining. The results showed that the length of colonic villi in the model group was significantly longer than that in the TAM group (636.01 ± 25.87 vs. 450.94 ± 52.62px, *P*=0.01). In addition, a large number of inflammatory cells were observed to have infiltrated the mucosa in the TAM group (**Figure 9**). These findings suggest that TAM could induce mucosal barrier damage in tumor-bearing mice.

## 4 Discussion

The human colorectum contains a vast microbiota that can affect various host physiological functions, including energy homeostasis, nutritional intake, and immune balance (23). Gut bacteria express different proteins, such as lipopolysaccharide (LPS) or flagellin, which can activate inflammatory responses by interacting with host receptors like TLR4 and TLR5, and regulate host systemic immunity (24). Furthermore, mounting evidence suggests that alterations in gut microbiota are associated with the development of breast cancer (25). Tamoxifen is an effective anti-tumor medication in ER+ breast cancer. It has been reported that tamoxifen treatment reduces the risk of invasive breast cancer by 49% in women at elevated risk (26). However, conflicting evidence has been presented regarding whether tamoxifen therapy increases the risks of receptor-negative contralateral breast cancer (6, 7). Establishing a tamoxifen-treated breast cancer model and analyzing its gut microbiota and inflammation may provide insights into tamoxifen-induced systematic alterations in breast cancer and identify potential intervention targets to reduce tamoxifen-related side effects.

Tamoxifen is an anti-estrogen medication that competitively inhibits estrogen by binding to ER in breast tumor cells. When orally administered, tamoxifen undergoes hepatic conversion into active metabolites, such as 4-hydroxytamoxifen (4HT), which compete with endogenous sex steroid hormones, such as estrogens, for binding to intracellular receptors, estrogen receptor alpha (ERα) and beta (ERβ). Tamoxifen can have adverse effects, including an increased risk of endometrial cancer, pulmonary embolism, stroke, and deep vein thrombosis (26). Estrogen receptors are not limited to breast or reproductive tissues, as a variety of tissues, including the intestine, brain, bone, and adipose tissue, also express estrogen receptors (27). It has been reported that representative orders, such as Lactobacillales, and specific phyla, such as Proteobacteria, Bacteroidetes, and Firmicutes, differ as a function of murine ERβ status, suggesting that ER status may play an important role in microbiota maintenance (28). Estrogen levels have been associated with alterations in gut microbiota (29). A clinical study suggested that indexes of bacterial diversity and the abundance of several bacterial genera were negatively correlated with estradiol levels (30). Estrogen can modify gut epithelial barrier integrity in mice, as evidenced by the observation that females are more resistant to gut injury than their male counterparts, and administration of estradiol to male rats mitigates gender differences (31). It has been reported that the gut microbiome mediates the preventive effect of 17β-estradiol against metabolic endotoxaemia and low-grade chronic inflammation: male and ovariectomized female mice have increased Proteobacteria and lipopolysaccharide (LPS) biosynthesis than normal female mice, and 17β-estradiol treatment decreased these to similar levels as female mice. Estrogen or estrogen-like compounds can decrease the LPS produced by the gut microbiome and gut permeability, resulting in reduced metabolic endotoxaemia (32). In this study, tamoxifen-related gut microbiota alterations were observed, along with damaged gut epithelial barrier and increased inflammation levels, which is consistent with earlier studies (4, 5, 33). A possible explanation for the changes observed in the gut microbiome is that tamoxifen had a direct effect on the gut microbiome, but this seems unlikely due to the fact that the ER gene evolved in vertebrates as well as some insects, and that ER activity has not been described in bacteria. It is more likely that tamoxifen’s effects on host ER resulted in a damaged gut epithelial barrier, increased inflammation levels, and changes in the gut microbiome.

Tamoxifen may also impact microbiota alteration and inflammation through changes in TLR5 expression. Studies have reported that elevated estrogen levels induce the downregulation of TLR5 expression (34), which is the only receptor of extracellular flagellin, an essential component of microbiome motility that initiates innate immune responses when recognized by TLR5 (35).. TLR5 deficient mice exhibited enriched fecal flagellin and altered gut microbiome, indicating impaired ability to inhibit flagellin-expressing, motility taxa (36)..

In our study, we observed that mRNA levels of Tlr5 in the colon, as well as inflammation biomarkers Il-6 and Tnf-α, were decreased in the model group and increased in the TAM group. Western blot analysis showed that TLR5 and IL-6 expression were decreased in the colon of the model group. Previous studies have reported that flagellin induced activation of NF-κB *via* TLR5 (37), which upregulates IL-6 (38).. Additionally, microbiome phenotype prediction indicated an enriched phenotype Contains_Mobile_Element in the model group, indicating impaired ability to inhibit motility taxa in mice of the model group, which was restored after tamoxifen treatment. Flagellin is the most important mobile element of bacteria. Thus, we assumed that the decreased TLR5 expression in the model group induced the enriched phenotype Contains_Mobile_Element. Gut microbiota alteration in the model and TAM groups may be induced by the effects of estrogen and tamoxifen on ER, respectively, and through the regulation of TLR5, thereby affecting TLR5-related host anti-bacteria immunity.

It is worth noting that the inflammatory conditions and gut microbiota composition can be impacted by the dose and duration of tamoxifen treatment. In clinical practice, different tamoxifen therapeutic regimens are adopted based on the clinical indication. For ERα-positive breast cancer, tamoxifen is prescribed at a daily dose of 20-40 mg for 5-year-long treatments. Conversely, ER-independent conditions, such as microbial infections or other ERα-negative oncological or fibrotic diseases, are treated with short-term therapies at doses ranging from 250-500 mg (39, 40). Tamoxifen induces “off-target” responses mediated by ERα-unrelated, low-affinity effectors that have been described in various cell lineages and physio-pathological conditions. Candidate mediators include PKC (protein kinase C), the transcription factors PPARγ (peroxisome proliferator-activated receptor gamma), GR (glucocorticoid receptor), STAT1 (signal transducer and activator of transcription 1), NRF2 (nuclear factor erythroid 2-related factor 2), as well as other undefined targets that regulate calcium homeostasis or lipid and sphingolipid metabolism.

Altered gut microbiota may, in turn, play a role in inflammation. As revealed by correlation-based clustering analysis, the abundance of certain group-characteristic taxa showed a strong association with mRNA levels of inflammation biomarkers in the colon. For example, *Lachnospiraceae-UCG-006*, a characteristic taxon of the model group, was negatively correlated with Tlr5. Increased *Lachnospiraceae* has been previously observed in TLR5-deficient mice (41). Lower levels of *Lachnospiraceae* have been independently associated with several chronic inflammatory diseases, including liver cirrhosis and inflammatory bowel disease (IBD) (42). Another characteristic taxa of the model group, *Anaerotruncus*, which negatively correlated with IFN-γ and IL-10, has been reported to be higher in individuals on a high saturated fatty acid diet and may be related to the development of specific diseases, such as pro-inflammatory diseases in women (43), and non-alcoholic fatty liver disease associated with hepatocellular carcinoma (44). In this study, *Prevotellaceae_UCG_001*, a characteristic taxon of the control and TAM groups, was positively correlated with inflammation biomarkers in the colon. It has been previously reported to be elevated in the colon of AOM/DSS-treated mice, and the effects were enhanced in ERβ KO mice (45). *Akkermansia*, another characteristic taxon of the control and TAM groups, was positively correlated with inflammation biomarkers in the colon. It has been associated with the protective mucus lining of the intestines, degrading host mucin into short-chain fatty acids that regulate the host’s biological functions. Decreased *Akkermansia* has been correlated with higher rates of obesity, increased symptoms of type 2 diabetes, and elevated inflammation levels (46). In obese women with breast cancer, *Akkermansia* may mediate the effects of dietary fiber in improving microbiome composition (47). Oral administration of *A. muciniphila* has shown significant improvement in symptoms in DSS-induced acute colitis (48), and it has been found to attenuate colitis-associated tumorigenesis by reducing infiltrating macrophages and CD8 cytotoxic T lymphocytes in the colon (49). *Bacteroides*, a characteristic taxon of the TAM group, was positively correlated with inflammation biomarkers in the colon and is known as an inflammation-promoting taxa. It has been reported as a major initiator and promoter of colorectal cancer as well as breast cancer (50) (51).

Nonetheless, studying human breast cancer through xenograft mouse models is extremely challenging, not only because of the differences between mice and humans but also due to the impaired immune system of nude mice. Additionally, the inflammation biomarkers in the serum showed less significance compared to those in the colon. Moreover, for the simplicity of the study design, estrogen tablets were used in the model and TAM groups but not in the control group, and antibiotic treatment aimed at eliminating the gut microbiota was not included. In future studies, a no-tumor control group with estrogen tablets or an exogenous estrogen-independent breast cancer model should be established, and antibiotic-treated groups should be included. Although this study has some limitations, it opens up new possibilities for exploring the associations between gut microbiota and inflammation in breast cancer.

## 5 Conclusion

This study suggests that estrogen treatment can lead to changes in the gut microbiota and reduced inflammation in a BC xenograft mice model. However, tamoxifen treatment resulted in a re-alteration of the gut microbiota and increased inflammation, possibly due to tamoxifen-induced damage to the intestinal epithelial barrier and up-regulation of TLR5.

## Data availability statement

The datasets presented in this study can be found in online repositories. The names of the repository/repositories and accession number(s) can be found below: https://www.ncbi.nlm.nih.gov/, PRJNA922201.

## Ethics statement

The animal study was reviewed and approved by the Institutional Animal Care and Use Committee of Zhejiang Chinese Medical University.

## Author contributions

HL, XG, and QZ contributed to the design and conception of the study. HL, MW, CX, QY, YJ and JS did the investigation. HL, XG, YC, and QZ wrote the manuscript. HL and QZ created tables and figures. HL, XG, and QZ guided manuscript writing and revised the manuscript. XG and QZ provided financial support. All authors contributed to the article and approved the submitted version.
