# Supplementary material for: Potential risk of tamoxifen: gut microbiota and inflammation in mice with breast cancer
Source: Front Oncol. 2023 Jul 4;13:1121471. doi: 10.3389/fonc.2023.1121471 (PMC10353877; doi:10.3389/fonc.2023.1121471)
Supplement: Supplementary file 1 [file Table_1.docx]

Supplementary Material

Potential Risk of Tamoxifen: Gut Microbiota and Inflammation in Mice with Breast Cancer

Hailong Li^1†^, Xiufei Gao^2†^, Yian Chen^3^, Mengqian Wang^3^, Chuchu Xu^3^, Qinghong Yu^3^, Ying Jin^3^, Jiaqing Song^3^, Qi Zhu^2*^

*** Correspondence:** Qi Zhu: zqwin12@163.com

TABLE S1 Primer sequence

| gene | Forward Primer Sequence | Reverse Primer Sequence |
| --- | --- | --- |
| *Gapdh* | CGGATTTGGTCGTATTG | GAAGATGGTGATGGGATT |
| *Il6* | GAGACTGGGGATGTCTGTAGC | TCACCAGCATCAGTCCCAAG |
| *Tlr5* | TCTCCTGGCTCAACCAAACC | GGCTTCCTCTTCATCGCAGT |
| *Tnfα* | CAGGCGGTGCCTATGTCTC | CGATCACCCCGAAGTTCAGTAG |
| *Il17a* | TCAGCGTGTCCAAACACTGAG | CGCCAAGGGAGTTAAAGACTT |
